# Hospitalisation Is Prognostic of Survival in Chronic Thromboembolic Pulmonary Hypertension

**DOI:** 10.3390/jcm11206189

**Published:** 2022-10-20

**Authors:** Pavel Jansa, David Ambrož, Michael Aschermann, Vladimír Černý, Vladimír Dytrych, Samuel Heller, Jan Kunstýř, Jaroslav Lindner, Aleš Linhart, Matúš Nižnanský, Michal Paďour, Tomáš Prskavec, Michal Širanec, Susan Edwards, Virginie Gressin, Matyáš Kuhn, Lilla Di Scala

**Affiliations:** 1 2nd Department of Internal Medicine–Department of Cardiovascular Medicine, First Faculty of Medicine, Charles University and General University Hospital, 128 08 Prague, Czech Republic; david.ambroz@vfn.cz (D.A.); aschermann@seznam.cz (M.A.); vladimirdytrych@seznam.cz (V.D.); shel@lf1.cuni.cz (S.H.); ales.linhart@vfn.cz (A.L.); michal.padour@vfn.cz (M.P.); michal.siranec@vfn.cz (M.Š.); 2 Department of Radiology, First Faculty of Medicine, Charles University and General University Hospital, 128 08 Prague, Czech Republic; vladimir.cerny@vfn.cz; 3 Department of Anesthesiology and Intensive Care, First Faculty of Medicine, Charles University and General University Hospital, 128 08 Prague, Czech Republic; jan.kunstyr@vfn.cz; 4 2nd Department of Surgery, Department of Cardiovascular Surgery, First Faculty of Medicine, Charles University and General University Hospital, 128 08 Prague, Czech Republic; lindner@seznam.cz (J.L.); matus.niznansky@vfn.cz (M.N.); tomas.prskavec@vfn.cz (T.P.); 5 Actelion Pharmaceuticals Ltd., A Janssen Pharmaceutical Company of Johnson & Johnson, 4123 Allschwil, Switzerland; sedwar10@its.jnj.com (S.E.); vgressin@its.jnj.com (V.G.); ldiscala@its.jnj.com (L.D.S.); 6 Data Analysis Department, Institute of Biostatistics and Analysis Ltd., 602 00 Brno, Czech Republic; kuhn@biostatistika.cz

**Keywords:** pulmonary hypertension, mortality, prognosis, CTEPH-related morbidity, hospitalisation, pulmonary endarterectomy

## Abstract

This analysis investigated the prognostic value of hospitalisation in chronic thromboembolic pulmonary hypertension (CTEPH) using data from the Czech Republic, wherein pulmonary endarterectomy (PEA) was the only targeted treatment option until 2015. Using a landmark method, this analysis quantified the association between a first CTEPH-related hospitalisation event occurring before 3-, 6-, 9-, and 12-month landmark timepoints and subsequent all-cause mortality in adult CTEPH patients diagnosed between 2003 and 2016 in the Czech Republic. Patients were stratified into operable and inoperable, according to PEA eligibility. CTEPH-related hospitalisations were defined as non-elective. Hospitalisations related to CTEPH diagnosis, PEA, balloon pulmonary angioplasty, or clinical trial participation were excluded. Of 436 patients who survived to ≥3 months post diagnosis, 309 were operable, and 127 were inoperable. Sex- and age-adjusted hazard ratios (HRs) showed CTEPH-related hospitalisation was a statistically significant prognostic indicator of mortality at 3, 9, and 12 months in inoperable patients, with an approximately 2-fold increased risk of death in the hospitalisation group (HRs [95% CI] ranging from 1.98 [1.06–3.70] to 2.17 [1.01–4.63]). There was also a trend of worse survival probabilities in the hospitalisation groups for operable patients, with the difference most pronounced at 3 months, with a 76% increased risk of death (adjusted HR [95% CI] 1.76 [1.15–2.68]). This first analysis on the prognostic value of CTEPH-related hospitalisations demonstrates that a first CTEPH-related hospitalisation is prognostic of mortality in CTEPH, particularly for inoperable patients. These patients may benefit from medical and/or interventional therapy.

## 1. Introduction

Chronic thromboembolic pulmonary hypertension (CTEPH) is a rare and life-threatening complication of acute pulmonary embolism [1,2,3]. The treatment of choice for eligible CTEPH patients is pulmonary endarterectomy (PEA) since it can achieve near-normal haemodynamics [1] and is associated with improved survival rates [4,5]. However, PEA is not feasible in all patients either for technical (distal disease) or medical reasons (unfavourable risk-benefit ratio of PEA or patient’s refusal) [1,6,7]. In these inoperable patients, management relies on medical therapy, such as riociguat [6] with or without balloon pulmonary angioplasty (BPA), an interventional procedure [2,6]. The 2015 European Society of Cardiology (ESC)/European Respiratory Society (ERS) guidelines recommend multimodal treatment, which combines surgical, interventional, and medical therapy [1]. This approach was reinforced at the 6th World Symposium on Pulmonary Hypertension [6] and in the 2020 ERS consensus statement on CTEPH [2], and it has now been confirmed in the 2022 ESC/ERS guidelines [8].

A better understanding of prognostic factors could help improve CTEPH management, particularly for those who do not benefit from PEA. In pulmonary arterial hypertension (PAH), PAH-related morbidity has been shown to be prognostic for subsequent survival using data from the event-driven SERAPHIN and GRIPHON clinical trials [9]. The analysis of these two trials used a landmarking-based method [10,11] to assess the prognostic value of morbidity events prior to the landmark timepoints on the risk of subsequent mortality. A prior PAH-related hospitalisation event was a strong prognostic indicator of mortality at landmark months 3, 6, and 12 of the GRIPHON trial, with hazard ratios (HRs) indicating a 3- to 6-fold increased risk of mortality for patients who experienced a PAH-related hospitalisation compared with those who did not [10]. However, the prognostic value of CTEPH-related hospitalisations for earlier mortality has not, to our knowledge, been investigated in CTEPH.

The objective of this current analysis was to investigate the prognostic role of CTEPH-related hospitalisation for the survival of patients in the Czech registry of CTEPH patients using the same landmark method described above. PEA was the only treatment option available to this population until 2015, when both riociguat and BPA became available. Importantly, diagnosis and management of CTEPH are centralised in a single expert centre in Prague, and comprehensive data on hospitalisations and vital status are available from national governmental registry databases [12].

## 2. Materials and Methods

### 2.1. Patients

This observational cohort from the General University Hospital in Prague is described in detail elsewhere [12]. Briefly, this registry included adults (≥18 years) with Czech citizenship who were newly diagnosed with CTEPH at the General University Hospital in Prague between 1 January 2003 and 31 December 2016. Observation continued until 31 December 2018. Diagnostic criteria were mean pulmonary arterial pressure (mPAP) ≥ 25 mm Hg; mismatched perfusion defects on lung ventilation perfusion scintigraphy; and imaging results consistent with CTEPH (diagnostic signs for CTEPH found using multi-detector computed tomography pulmonary angiography or conventional pulmonary angiography). Only patients who survived until the first 3-month landmark were included in the present analyses. The study was approved by the Prague General University Hospital’s research ethics committee. As this was a retrospectively defined observational cohort, and only anonymous data were processed (including mortality data, which were received in anonymised form), individual informed consent was not required.

### 2.2. Study Design and Outcome Measures

A landmark analysis was applied to retrospective data from this registry. This landmark analysis quantifies the association of a first hospitalisation event occurring before the landmark timepoint with a subsequent all-cause mortality event [10]. Hospitalisations subsequent to the first are not included in this analysis. Hospitalisation and vital status data were extracted from national registries led by the Institute of Health Information and Statistics of the Czech Republic (IHIS CR) [13].

CTEPH-related hospitalisations were defined as non-elective and excluded hospitalisations due to CTEPH diagnosis, PEA, BPA, or clinical trial participation. PEA-related hospitalisations were those that occurred at the same time as PEA and/or started or ended within 1 day before or after PEA. BPA-related hospitalisations were those that occurred at the same time as BPA. Hospitalisation due to clinical trial participation included all hospitalisations that occurred at the same time as study protocol procedures that required hospitalisation. CTEPH-related hospitalisations were identified based on International Statistical Classification of Diseases (ICD) codes, and each reason (ICD code) was reviewed and adjudicated by Prof. P. Jansa (Department of Cardiovascular Medicine, General University Hospital, Prague, Czech Republic) to ensure CTEPH-related hospitalisations were correctly identified.

Each patient was followed from CTEPH diagnosis to death, loss to follow-up, or 31 December 2018 (data cut-off), whichever came first. The index date analysis was the date of CTEPH diagnosis confirmation, and the landmarking timepoints were: 3, 6, 9, and 12 months after CTEPH diagnosis.

In the main analysis, patients were stratified into operable and inoperable groups according to whether they were eligible for PEA at index date. Patients’ operability status was determined at diagnosis by an interdisciplinary team including a PEA surgeon, pulmonary hypertension (PH) specialist, cardiac anaesthesiologist, and radiologist. Inoperable patients were those who were technically inoperable (due to distal disease). Not all operable patients were operated, as some were medically inoperable, due to either an unfavourable benefit-risk ratio (patients considered unfit for surgery or had comorbidities or other contributors to symptoms, in addition to clot burden, that precluded PEA) or patient refusal.

In a supplementary landmark analysis, patients were stratified into operated (those who underwent PEA surgery during the observation period) and not-operated. These two cohorts overlap with operable and non-operable cohorts. Operable patients were further stratified into operable and already operated patients (hereafter termed “already-operated patients”) and those who had not yet undergone PEA (“not-currently-operated patients”).

Cause of death was reported for all patients included in this analysis (those who survived to ≥3 months post diagnosis), by operability status and by whether they were operated on. Cause of death was identified from ICD codes in the national death registry, and each reason (ICD code) was reviewed and adjudicated by Prof. P. Jansa (Department of Cardiovascular Medicine, General University Hospital, Prague, Czech Republic).

### 2.3. Statistical Analysis

Survival was estimated based on Kaplan–Meier curves. All patients reaching the landmark timepoint were evaluated for survival up to death, loss to follow-up, or data cut-off (31 December 2018). If a patient was alive at the end of follow-up, their survival was censored. Kaplan–Meier curves are cut at the point when less than 10% of patients are at risk [14]. A Cox proportional hazard model (model M1, not adjusted) was used to assess the HR for patients with CTEPH-related hospitalisation versus patients with no hospitalisation at defined timepoints (patients with no hospitalisation were used as reference). Adjusted HRs were also computed using another model (M2) adjusted by age at diagnosis and sex. Forest plots for visualisation of models, and computed HRs were adopted. HRs were reported for subgroups only if they had at least 50 observations and at least 10 events per group (this is a subjective cut-off that maintains the interpretability of results).

## 3. Results

### 3.1. Patient Characteristics

In total, 453 patients were diagnosed between 2003 and 2016 and followed up for a median of 6.1 (range 0; 16) years. The median age at diagnosis was 65.2 (range 19–85) years, and 45.5% of patients were female [12]. During observation, 236 patients (52.1%) underwent PEA; the median time from diagnosis to PEA was 2.9 (range 0.0–101.2) months. Most (65.9%) of the 208 patients who were assessed had no residual PH 6 months after PEA. Sixty-eight (28.8%) operated patients died, and of these, twenty-three (33.8%) died within 3 months of PEA. Two hundred and seventeen patients did not undergo PEA, most (59.4%) due to distal disease. Most had severe disease at diagnosis as shown by the New York Heart Association functional classification (NYHA FC), 6-minute walk distance (6MWD), and haemodynamic parameters [12,15].

Overall, 436 patients survived until ≥3 months post diagnosis and were included in this analysis. Of those, 309 were operable, of whom 229 underwent PEA (operated patients), while 127 patients were inoperable, and 207 patients did not undergo PEA (not-operated patients). These groups were overlapping (Figure S1). For the landmark analysis, each of these four cohorts was further stratified by whether patients had experienced a first hospitalisation event prior to the 3-, 6-, 9,m and 12-month landmarks. The characteristics of these patient subgroups at diagnosis are shown in Table 1 (inoperable), Table 2 (operable), Table S1 (not-operated), and Table S2 (operated).

### 3.2. Landmark Analysis: Operable vs. Inoperable

**Figure 1:**
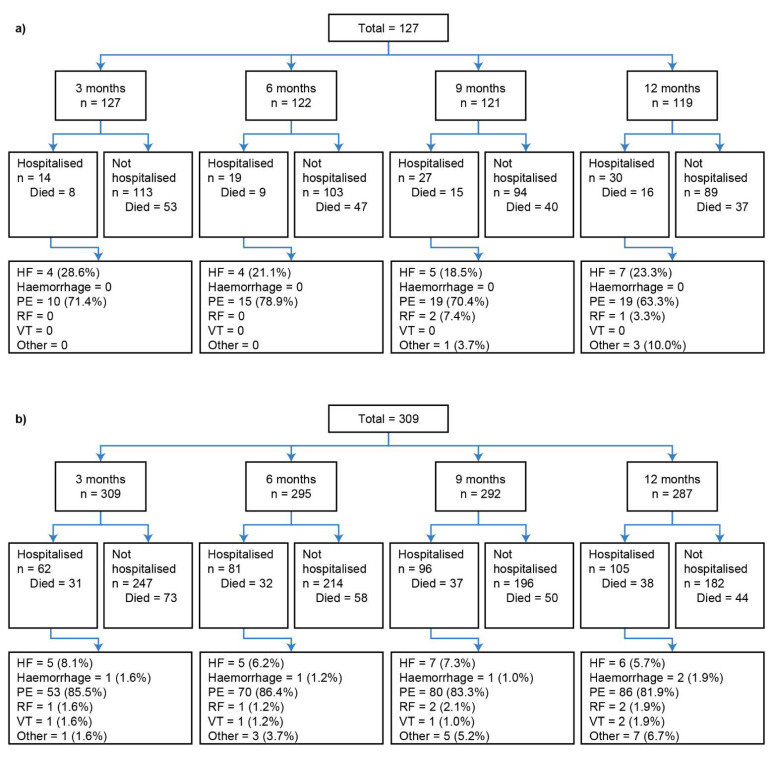
Landmark analysis patient disposition for (**a**) inoperable and (**b**) operable patients. The number of patients listed as “Died” in each group refers to the status at the data cut-off of 31 December 2018; all patients who did not die by this date were classified as “censored”. Percentages may not total to 100%, as the denominator is number of patients; however, patients may have had more than one hospitalisation. Reasons for hospitalisation are summarised in the final row. HF, heart failure; PE, pulmonary embolism; RF, respiratory failure; VT, venous thromboembolism.

**Figure 2:**
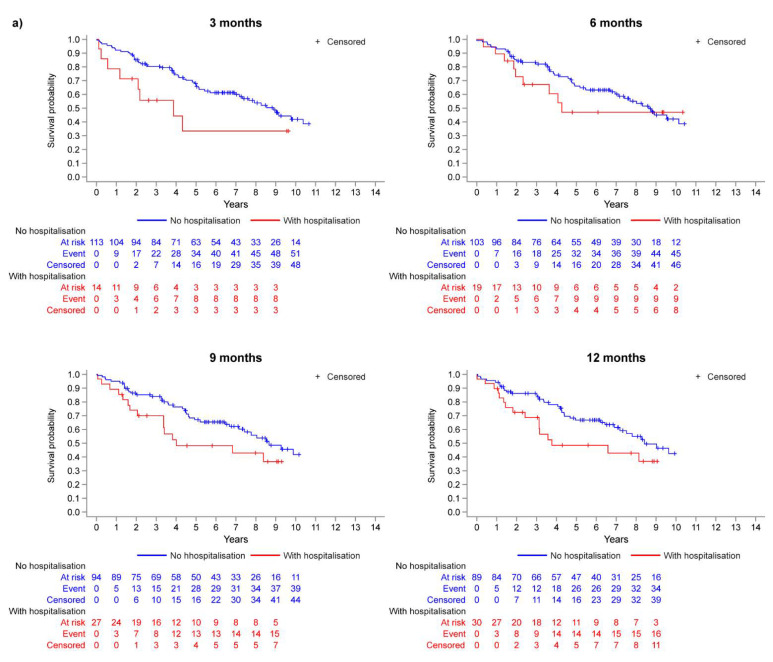

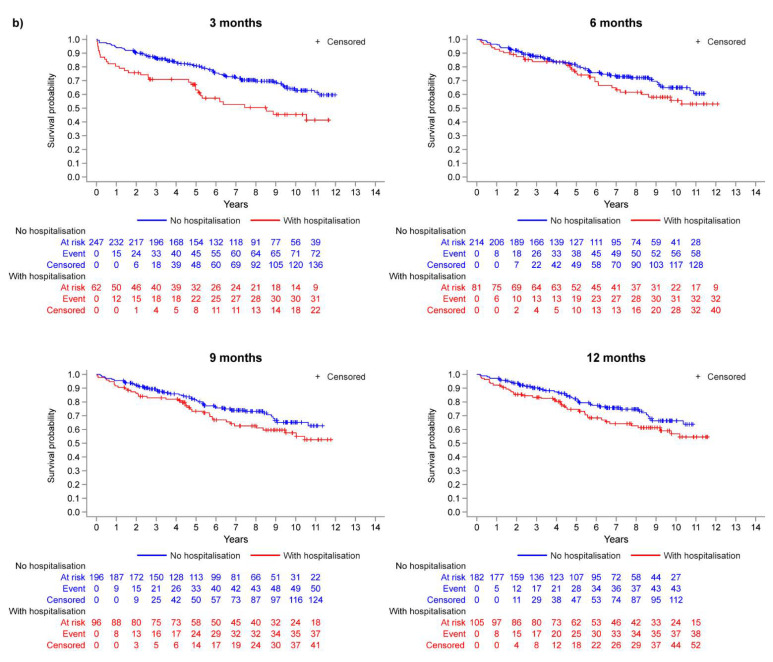
Kaplan–Meier analysis: survival probability at each landmark timepoint (model M1) for (**a**) inoperable patients and (**b**) operable patients.

**Figure 3:**
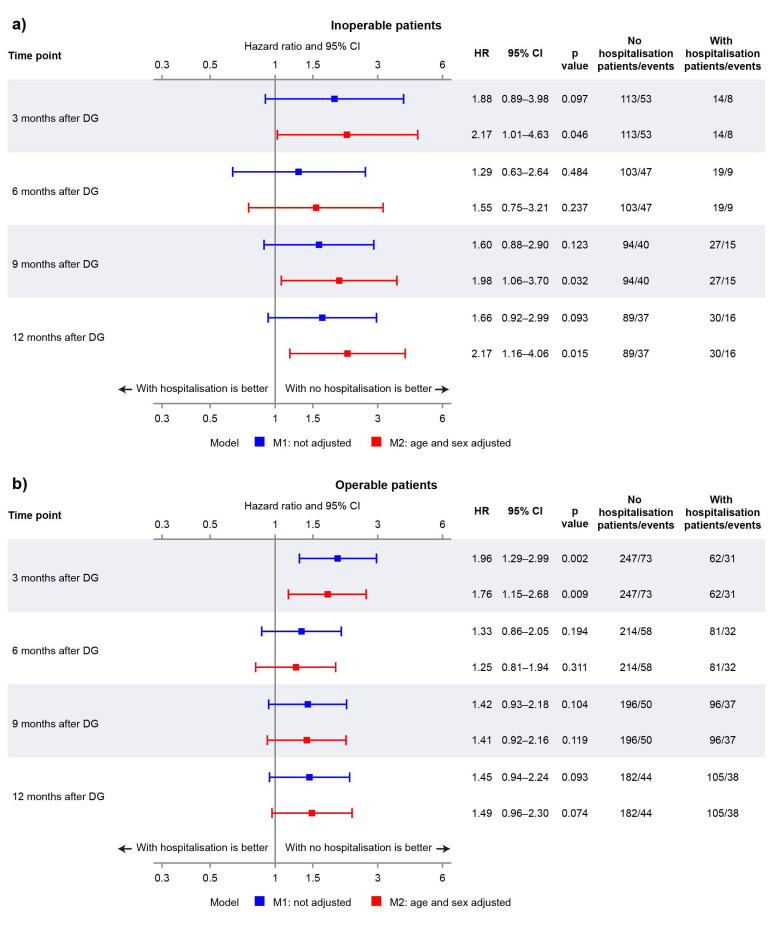
Forest plot showing hazard ratios (HRs) for non-adjusted and adjusted models at landmark timepoints for (**a**) inoperable patients and (**b**) operable patients.

The disposition of the inoperable group is summarised in Figure 1a. Of those who reached month 3, 6, 9, and 12, the percentages of patients who experienced a first hospitalisation event prior to each respective landmark timepoint were 11.0%, 15.6%, 22.3%, and 25.2% (Figure 1a). The Kaplan–Meier curves in Figure 2a illustrate the trend for worse survival in patients who experienced a first CTEPH-related hospitalisation across all landmark timepoints. Sex- and age-adjusted HRs show a statistically significant and approximate 2-fold increased risk of death in the hospitalisation group (versus no hospitalisation) at 3, 9, and 12 months (Figure 3a).

In the operable group, 20.1%, 27.5%, 32.9%, and 36.6% of patients who reached months 3, 6, 9, and 12 experienced a first hospitalisation event (Figure 1b). There was a trend for worse survival probabilities with hospitalisation, and the difference was more pronounced at the month 3 landmark (Figure 2b), with a 76% increased risk of death compared with those who were not hospitalised by month 3 (Figure 3b). The risk of death was not statistically significantly different between the hospitalisation and no-hospitalisation groups at later landmarks (Figure 3b).

Table S3 shows the cause of death for all the patients used in this analysis (those who survived to the 3-month landmark timepoint) by operability status. The cause of death is based on ICD codes from the national death registry. Pulmonary embolism was the most common cause of death, as identified by ICD codes. However, it is important to note that there were no CTEPH-specific ICD codes available until October 2017 [16]. Therefore, “pulmonary embolism” likely reflects progression of CTEPH rather than a subsequent acute PE. The next most common causes of death included the comorbidities of ischaemic heart disease (in operable, inoperable [Table S3], and not-operated cohorts [Table S4]) followed by malignancy (in operable, inoperable [Table S3], and operated patients [Table S4]).

### 3.3. Landmark Analysis: Operated vs. Not-Operated

Patient disposition for the operated and not-operated cohorts is shown in Figure S2, and the cause of death among these cohorts is summarized in Table S4. Among not-operated patients, an increased risk of death (between 58% and 144% increase) was observed in those who experienced a first hospitalisation event prior to the landmark timepoints (Figures S3a and S4a). All HRs calculated, except for the 6-month unadjusted analysis, were statistically significant (Figure S4a). The same trend was observed in operated patients, with the increased risk of death for the hospitalised group being between 35% and 93% although only the 3-month landmark result was statistically significant (Figures S3b and S4b). To note, this analysis is impacted by immortal time bias, as patients who were hospitalised and then died before being operated on were not included in the operated group.

### 3.4. Landmark Analysis of Operable Subgroups: Already-Operated vs. Not-Currently-Operated

Operable patients were stratified into those already-operated versus those not-currently-operated, and this analysis involved very few events. The not-currently-operated findings were similar to those of the overall operable group; a trend for increased risk of death with hospitalisation was observed (Figure S5), and the difference was statistically significant at the 3-month landmark (Figure S6a). Prior hospitalisation events appeared to have very little effect on mortality in the already-operated patients at the 3-month landmark although HRs for all later landmarks were in favour of no hospitalisation (Figures S6b and S7).

## 4. Discussion

To our knowledge, this landmark analysis of a national registry is the first to assess the prognostic value of CTEPH-related hospitalisations on mortality. Across all patient cohorts and landmark points, there was a consistent trend for increased mortality associated with CTEPH-related hospitalisation, demonstrating the importance of preventing disease progression in CTEPH. Moreover, the data from inoperable patients show a significant increase in mortality risk by adjusted HRs and Kaplan–Meier analysis when patients experienced a first CTEPH-related hospitalisation within 3, 9, and 12 months after diagnosis.

The magnitude and statistical significance of HRs differed according to the patients’ operability status, whether patients ultimately underwent PEA, and the landmark timepoint. Regarding operability status, there was a stronger indication that hospitalisation is prognostic in inoperable patients (versus operable), highlighting the importance of preventing hospitalisations in this cohort. As such, these patients may benefit from active treatment with BPA and/or medical therapy though further studies are required. Moreover, there is currently no consensus on which patients would benefit most from each of the combinations of multimodal therapy [2,8], and research could investigate which patient cohorts would be good candidates for certain combinations. Of note, the percentage of patients who were hospitalised was higher in the operable group (versus inoperable). This finding may be related to the study definition of CTEPH-related hospitalisation: PEA-related hospitalisations could have occurred outside the window used in the definition (starting or ending within 1 day before or after PEA) and contributed to CTEPH-related hospitalisations.

The prognostic value of hospitalisations was greater in not-operated patients compared with operated patients, who benefitted from PEA and may be less likely to experience a hospitalisation with a fatal outcome. However, it is worth repeating that this analysis could be impacted by immortal time bias. Operable patients were further sub-grouped: findings from not-currently-operated patients were similar to those from the overall operable group (trend for lower survival probability in hospitalised patients, with the 3-month landmark showing the strongest signal), whereas in already-operated patients, the same trend was observed for all except the 3-month landmark. The lack of effect at 3 months could be due to hospitalisations in these patients representing PEA-related complications and/or the most severe patients dying shortly after PEA (one-third of operated patients who died did so within 3 months of PEA).

The largest HRs were consistently observed at the 3-month landmark. This is the earliest timepoint, and therefore, the number of operable/operated patients who have undergone PEA is lower compared with later timepoints, and this landmark is less affected by the benefit of PEA. The impact of immortal time bias on the operated cohort is also likely to be smaller at the earliest landmark. The lower HRs in later landmarks may also be due to the death of patients with the most severe condition within 3 months of diagnosis, with the number of patients at risk becoming smaller as more patients die over time. This makes the interpretation of these data less straightforward. There may also be other factors that change between landmark timepoints that this analysis does not capture (e.g., subsequent hospitalisations, decline in general health).

Data from PH clinical trials also highlight the importance of preventing hospitalisations [17,18,19,20] though there is more evidence in PAH than in CTEPH. For example, in the GRIPHON trial of PAH patients, the treatment effect (selexipag versus placebo) for the primary composite end-point of PAH-related morbidity or mortality (HR 0.60 [99% CI 0.46–0.78], *p* < 0.001) was driven by differences in PAH-related hospitalisation (47% of worsening events) and disease progression (35% of events) [21]. In CTEPH, the number of clinical worsening events in the CHEST-1, BENEFiT, MERIT-1, and CTREPH trials were low in each treatment arm, and no significant treatment differences were observed [18,19,20,22]. However, it should be noted that these are short-term trials with relatively small sample sizes [18,19,20,22] compared with event-driven trials, such as GRIPHON [21], and that the definition of clinical worsening (and its hospitalisation component) differs across trials. The ongoing event-driven MACiTEPH trial of macitentan 75 mg (versus placebo) in inoperable or recurrent CTEPH includes time to clinical worsening up to the end of double-blind treatment as the key secondary end-point and captures unplanned PH-related hospitalisation as part of this composite outcome [23]. The double-blind treatment period comprises an initial 28-week fixed-duration period and a subsequent period of variable duration depending on when patients entered the study, as patients remain on double-blind treatment until all participants have completed week 28 (primary end-point assessment) [23]. Therefore, this study design will better capture hospitalisations and clinical worsening than the shorter-term trials. The prognostic value of PH-related hospitalisations for mortality has been demonstrated for PAH in clinical trials [9] and in clinical practice [24]. The findings of the present real-world study suggest that a first CTEPH-related hospitalisation could be prognostic for mortality in CTEPH patients, particularly in inoperable patients.

The strengths of this study include the use of comprehensive hospitalisation and mortality data from the national databases, allowing a thorough assessment of the prognostic value of hospitalisations. All ICD codes for hospitalisation events were adjudicated by the lead author to ensure all events were related to CTEPH. These ICD codes had been assigned by the treating physician, and retrospective adjudication of clinical data was not possible. Another unique feature of this registry population is that it includes well-defined patient cohorts, as they have primarily had their CTEPH managed with PEA since neither riociguat nor BPA were available in the Czech Republic until 2015. As with all landmark analyses, the number of patients at risk is greater at earlier landmarks, and the number of patients who have died and are removed from analysis increases as time goes on. Additional limitations of this analysis include that (i) our analysis was only adjusted for age and sex but not other factors that are known to be prognostic in CTEPH; (ii) only first hospitalisation events were accounted for in this analysis; and (iii) the proportional hazard assumption was not always maintained through all timepoints, which suggests potential interference of time-varying effects. For the analysis of operated versus not-operated patients, the analysis does not capture whether PEA in operated patients took place before or after the landmark timepoints, and these data are impacted by immortal time bias.

## 5. Conclusions

This is the first study to assess the prognostic value of CTEPH-related hospitalisations on mortality. These data, from an era in which there was limited use of BPA and medical therapy for CTEPH, show that experiencing a non-elective CTEPH-related hospitalisation within 1 year of diagnosis is prognostic for worse survival in patients with this condition, particularly for inoperable patients. Inoperable patients may benefit from active treatment with medical therapy and/or BPA to prevent hospitalisations and potentially improve their outcomes.

## Figures and Tables

**Table 1:** Baseline characteristics of inoperable patients at landmark timepoints.

	Month 3 Landmark		Month 6 Landmark		Month 9 Landmark		Month 12 Landmark	
Parameter	Prior Hospitalisation Event (n = 14)	No Prior Hospitalisation Event (n = 113)	Prior Hospitalisation Event (n = 19)	No Prior Hospitalisation Event (n = 103)	Prior Hospitalisation Event (n = 27)	No Prior Hospitalisation Event (n = 94)	Prior Hospitalisation Event (n = 30)	No Prior Hospitalisation Event (n = 89)
Age, mean (SD), years	65.7 (10.04)	66.7 (13.05)	64.6 (13.11)	66.8 (12.89)	64.2 (12.53)	67.1 (13.06)	64.1 (12.80)	67.2 (13.12)
Sex, n (%), female	9 (64.3%)	61 (54.0%)	12 (63.2%)	56 (54.4%)	13 (48.1%)	55 (58.5%)	16 (53.3%)	51 (57.3%)
BMI, mean (SD), kg/m^2^ [n]	28.1 (5.67) [13]	28.3 (5.08) [105]	28.7 (5.11) [17]	28.4 (5.08) [97]	28.9 (4.49) [25]	28.4 (5.20) [88]	29.7 (4.03) [28]	28.2 (5.24) [84]
DVT history, n (%)	3 (21.4%)	40 (36.0%)	5 (27.8%)	37 (36.3%)	7 (26.9%)	35 (37.6%)	9 (31.0%)	32 (36.4%)
PE history, n (%)	10 (71.4%)	82 (72.6%)	11 (57.9%)	77 (74.8%)	18 (66.7%)	69 (73.4%)	21 (70.0%)	66 (74.2%)
Time from first PE to diagnosis, mean (SD), years	5.4 (8.22)	5.2 (7.03)	6.0 (7.55)	5.1 (7.20)	6.7 (7.76)	4.9 (7.09)	6.5 (7.63)	4.9 (7.10)
NYHA FC, n (%) [n]								
FC I/II	–	4 (4.0%) [101]	–	4 (4.3%) [92]	–	4 (4.8%) [84]	–	4 (5.1%) [79]
FC III/IV	12 (100%) [12]	97 (96.0%) [101]	16 (100%) [16]	88 (95.7%) [92]	24 (100%) [24]	80 (95.2%) [84]	27 (100%) [27]	75 (94.9%) [79]
6MWT, mean (SD), metres [n]	269.7 (104.9) [11]	337.8 (112.4) [82]	279.8 (133.5) [14]	343.8 (107.7) [75]	309.6 (127.7) [21]	341.1 (109.0) [68]	308.8 (121.7) [24]	348.0 (107.9) [63]
RHC								
mPAP, mean (SD), mmHg [n]	50.1 (12.60) [13]	43.2 (11.69) [108]	51.3 (14.65) [18]	42.5 (11.03) [99]	50.2 (12.89) [25]	42.0 (11.29) [91]	50.1 (13.03) [28]	41.5 (11.09) [86]
PVR, mean (SD), dyn s/cm^5^ [n]	905.1 (381.1) [12]	629.2 (325.5) [107]	822.6 (430.6) [17]	613.0 (311.6) [98]	765.3 (389.7) [24]	604.7 (311.8) [90]	716.4 (365.3) [27]	601.5 (314.6) [85]
CI, mean (SD), L/min/m^2^ [n]	2.0 (0.40) [12]	2.3 (0.58) [106]	2.3 (0.64) [17]	2.3 (0.56) [97]	2.3 (0.60) [24]	2.3 (0.56) [89]	2.4 (0.64) [27]	2.3 (0.54) [84]
BNP, mean (SD), pg/mL [n]	745.0 (475.7) [6]	284.4 (322.9) [49]	445.5 (312.1) [8]	289.4 (332.2) [46]	350.0 (276.1) [13]	300.6 (349.1) [41]	264.8 (251.4) [15]	294.0 (323.9) [37]
Anticoagulation, n (%)								
NOAC	1 (7.1%)	4 (3.5%)	1 (5.3%)	4 (3.9%)	1 (3.7%)	4 (4.3%)	1 (3.3%)	4 (4.5%)
Vitamin K antagonist	11 (78.6%)	99 (87.6%)	15 (78.9%)	91 (88.3%)	23 (85.2%)	82 (87.2%)	26 (86.7%)	78 (87.6%)
Other anticoagulants	2 (14.3%)	10 (8.8%)	3 (15.8%)	8 (7.8%)	3 (11.1%)	8 (8.5%)	3 (10.0%)	7 (7.9%)
Time to diagnosis from study start ^1^, mean (SD), years	8.91 (3.631)	7.66 (3.103)	8.02 (3.372)	7.78 (3.143)	7.69 (2.871)	7.79 (3.218)	7.98 (2.887)	7.68 (3.253)

^1.^ 1 January 2003. BNP, brain natriuretic peptide; CI, cardiac index; DVT, deep vein thrombosis; mPAP, mean pulmonary artery pressure; NOAC, non-vitamin K antagonist oral anticoagulants; NYHA FC, New York Heart Association functional class; PE, pulmonary embolism; PVR, pulmonary vascular resistance; RHC, right heart catheterisation parameters; SD, standard deviation; 6MWT, 6-minute walk test.

**Table 2:** Baseline characteristics of operable patients at landmark timepoints.

	Month 3 Landmark		Month 6 Landmark		Month 9 Landmark		Month 12 Landmark	
Parameter	Prior Hospitalisation Event (n = 62)	No Prior Hospitalisation Event (n = 247)	Prior Hospitalisation Event (n = 81)	No Prior Hospitalisation Event (n = 214)	Prior Hospitalisation Event (n = 96)	No Prior Hospitalisation Event (n = 196)	Prior Hospitalisation Event (n = 105)	No Prior Hospitalisation Event (n = 182)
Age, mean (SD), years	64.4 (10.59)	60.7 (12.65)	62.8 (11.54)	60.7 (12.84)	61.3 (12.08)	61.3 (12.72)	60.8 (13.06)	61.5 (12.28)
Sex, n (%), female	28 (45.2%)	100 (40.5%)	39 (48.1%)	84 (39.3%)	46 (47.9%)	76 (38.8%)	51 (48.6%)	70 (38.5%)
BMI, mean (SD), kg/m^2^ [n]	28.5 (6.08) [53]	28.4 (5.54) [208]	28.1 (5.12) [66]	28.5 (5.65) [184]	28.5 (5.27) [79]	28.3 (5.65) [168]	28.4 (5.30) [89]	28.4 (5.59) [154]
DVT history, n (%)	27 (45.8%)	106 (43.1%)	35 (44.3%)	93 (43.7%)	40 (42.6%)	85 (43.6%)	45 (43.7%)	79 (43.6%)
PE history, n (%)	50 (80.6%)	196 (79.4%)	66 (81.5%)	170 (79.4%)	75 (78.1%)	158 (80.6%)	84 (80.0%)	146 (80.2%)
Time from first PE to diagnosis, mean (SD), years	7.1 (8.70)	4.9 (7.19)	5.5 (6.54)	5.0 (7.49)	5.8 (7.77)	4.7 (6.94)	5.7 (7.57)	4.8 (7.07)
NYHA FC, n (%) [n]								
FC I/II	2 (3.2%) [62]	30 (12.2%) [246]	5 (6.2%) [81]	26 (12.2%) [213]	5 (5.2%) [96]	25 (12.8%) [195]	6 (5.8%) [104]	24 (13.2%) [182]
FC III/IV	60 (96.8%) [62]	216 (87.8%) [246]	76 (93.8%) [81]	187 (87.8%) [213]	91 (94.8%) [96]	170 (87.2%) [195]	98 (94.2%) [104]	158 (86.8%) [182]
6MWT, mean (SD), metres [n]	305.6 (102.3) [49]	354.0 (107.4) [217]	309.5 (94.79) [67]	361.4 (108.4) [186]	309.0 (98.60) [83]	365.9 (106.5) [169]	316.7 (102.4) [90]	366.5 (106.0) [157]
RHC								
mPAP, mean (SD), mmHg [n]	52.0 (12.97) [62]	47.8 (12.58) [240]	50.7 (12.50) [79]	47.5 (12.63) [209]	50.8 (12.30) [94]	47.1 (12.71) [191]	50.4 (12.45) [102]	47.0 (12.63) [178]
PVR, mean (SD), dyn s/cm^5^ [n]	802.2 (319.2) [62]	730.2 (347.3) [239]	807.3 (344.8) [79]	714.4 (333.0) [208]	783.9 (324.7) [94]	714.3 (341.4) [190]	780.4 (336.4) [101]	704.5 (319.7) [179]
CI, mean (SD), L/min/m^2^ [n]	2.2 (0.45) [61]	2.3 (0.53) [234]	2.2 (0.46) [78]	2.3 (0.52) [203]	2.2 (0.46) [93]	2.3 (0.52) [185]	2.2 (0.50) [101]	2.3 (0.50) [172]
BNP, mean (SD), pg/mL [n]	313.5 (251.7) [18]	392.9 (597.1) [113]	267.0 (234.1) [25]	397.8 (601.8) [102]	311.4 (341.4) [34]	395.8 (613.0) [92]	316.6 (349.4) [36]	373.8 (605.5) [88]
Anticoagulation, n (%) [n]								
NOAC	–	7 (2.9%) [244]	–	7 (3.3%) [214]	–	7 (3.6%) [196]	–	7 (3.8%) [182]
Vitamin K antagonist	47 (75.8%) [62]	208 (85.2%) [244]	68 (84.0%) [81]	179 (83.6%) [214]	81 (84.4%) [96]	164 (83.7%) [196]	90 (85.7%) [105]	150 (82.4%) [182]
Other anticoagulants	15 (24.2%) [62]	29 (11.9%) [244]	13 (16.0%) [81]	28 (13.1%) [214]	15 (15.6%) [96]	25 (12.8%) [196]	15 (14.3%) [105]	25 (13.7%) [182]
Time to diagnosis from study start ^1^, mean (SD), years	6.60 (3.253)	7.58 (3.434)	6.21 (3.179)	7.87 (3.395)	6.41 (3.273)	7.88 (3.393)	6.51 (3.472)	7.90 (3.333)

^1.^ 1 January 2003. BNP, brain natriuretic peptide; CI, cardiac index; DVT, deep vein thrombosis; mPAP, mean pulmonary artery pressure; NOAC, non-vitamin K antagonist oral anticoagulants; NYHA FC, New York Heart Association functional class; PE, pulmonary embolism; PVR, pulmonary vascular resistance; RHC, right heart catheterisation parameters; SD, standard deviation; 6MWT, 6-minute walk test.

## Data Availability

The data presented in this study are available on request from the corresponding author.

## Supplementary Materials

The following supporting information can be downloaded at: https://www.mdpi.com/2077-0383/11/20/6189/s1, Figure S1: Included patients (those who survived for at least 3 months post diagnosis) by operability status and whether they underwent PEA; Figure S2: Landmark analysis patient disposition for (a) not-operated (b) operated patients. All patients who did not die by the data cut-off on 31 December 2018 were classified as “censored”. Percentages may not total to 100%, as the denominator is number of patients; however, patients may have had more than one hospitalisation. Reasons for hospitalisation are summarised in the final row; Figure S3: Kaplan–Meier analysis: survival probability at each landmark timepoint (model M1) in (a) not-operated patients and (b) operated patients; Figure S4: Forest plot showing hazard ratios (HRs) for non-adjusted and adjusted models at landmark timepoints in (a) not-operated patients and (b) operated patients; Figure S5: Kaplan–Meier analysis in operable patients who were not-currently-operated patients: survival probability at each landmark timepoint (model M1); Figure S6: Forest plot showing hazard ratios (HRs) for non-adjusted and adjusted models at landmark timepoints in (a) operable patients who were not-currently-operated and (b) operable patients who were already-operated; Figure S7: Kaplan–Meier analysis in operable patients who were already-operated patients: survival probability at each landmark timepoint (model M1); Table S1: Baseline characteristics of not-operated patients at landmark timepoints; Table S2: Baseline characteristics of operated patients at landmark timepoints; Table S3: Cause of death in study cohort, by operability status; Table S4: Cause of death in study cohort, by whether patients were operated on.
