# Correction: Jansa et al. Hospitalisation Is Prognostic of Survival in Chronic Thromboembolic Pulmonary Hypertension. *J. Clin. Med.* **2022**, *11*, 6189

**DOI:** 10.3390/jcm12123939

**Published:** 2023-06-09

**Authors:** Pavel Jansa, David Ambrož, Michael Aschermann, Vladimír Černý, Vladimír Dytrych, Samuel Heller, Jan Kunstýř, Jaroslav Lindner, Aleš Linhart, Matúš Nižnanský, Michal Pad’our, Tomáš Prskavec, Michal Širanec, Susan Edwards, Virginie Gressin, Matyáš Kuhn, Lilla Di Scala

**Affiliations:** 1 2nd Department of Internal Medicine–Department of Cardiovascular Medicine, First Faculty of Medicine, Charles University and General University Hospital, 128 08 Prague, Czech Republic; 2 Department of Radiology, First Faculty of Medicine, Charles University and General University Hospital, 128 08 Prague, Czech Republic; 3 Department of Anesthesiology and Intensive Care, First Faculty of Medicine, Charles University and General University Hospital, 128 08 Prague, Czech Republic; 4 2nd Department of Surgery, Department of Cardiovascular Surgery, First Faculty of Medicine, Charles University and General University Hospital, 128 08 Prague, Czech Republic; 5 Actelion Pharmaceuticals Ltd, A Janssen Pharmaceutical Company of Johnson & Johnson, 4123 Allschwil, Switzerland; 6 Data Analysis Department, Institute of Biostatistics and Analysis Ltd, 602 00 Brno, Czech Republic

Error in Figure

Due to problems with Figure 1 and Figure 2 in the original publication [1], we replaced it with an updated version of Figure 1 and Figure 2.

The authors wish to replace Figure 1 with the latest version, which includes left aligning of the text in the boxes in the third row down and an indentation added to the “Died” lines, as requested by the peer reviewers.

**Figure 1:**
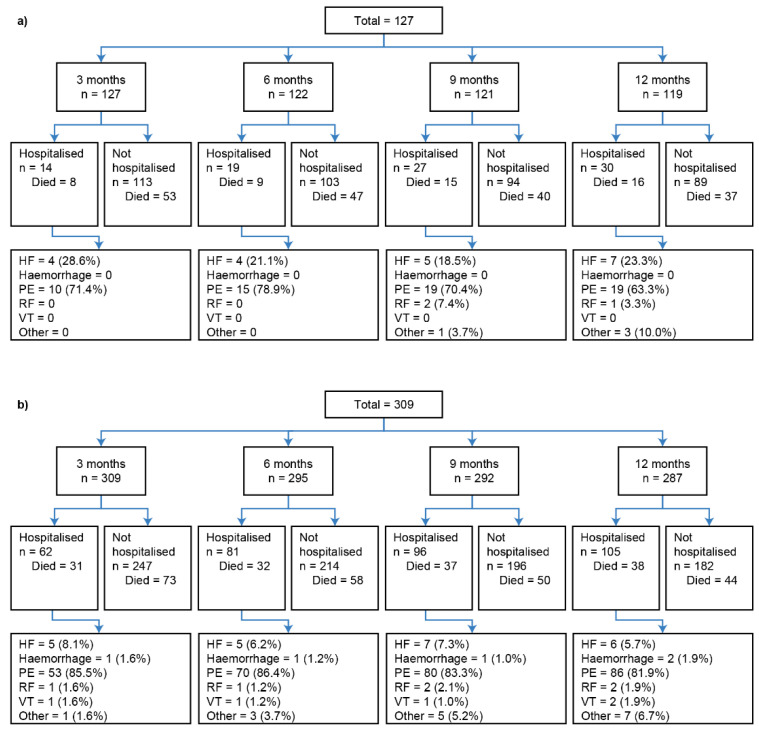
Landmark analysis patient disposition for (**a**) inoperable and (**b**) operable patients. The number of patients listed as “Died” in each group refers to the status at the data cut-off of 31 December 2018; all patients who did not die by this date were classified as “censored”. Percentages may not total to 100%, as the denominator is number of patients; however, patients may have had more than one hospitalisation. Reasons for hospitalisation are summarised in the final row. HF, heart failure; PE, pulmonary embolism; RF, respiratory failure; VT, venous thromboembolism.

The authors wish to replace Figure 2 with the correct version:

**Figure 2:**
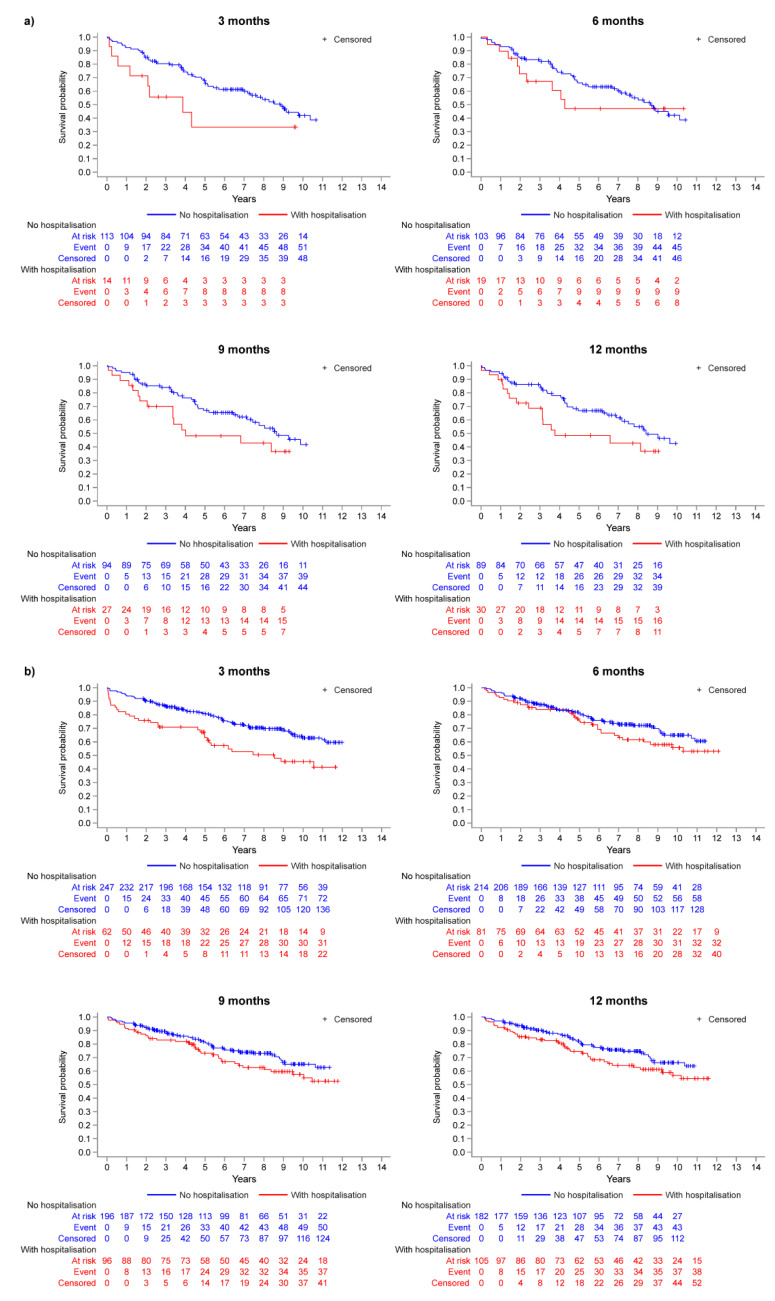
Kaplan–Meier analysis: survival probability at each landmark timepoint (model M1) for (**a**) inoperable patients and (**b**) operable patients.

The authors state that the scientific conclusions are unaffected. This correction was approved by the academic editor. The original publication has also been updated.
