# Supplementary material for: Hospitalisation Is Prognostic of Survival in Chronic Thromboembolic Pulmonary Hypertension
Source: J Clin Med. 2022 Oct 20;11(20):6189. doi: 10.3390/jcm11206189 (PMC9605547; doi:10.3390/jcm11206189)
Supplement: Supplementary file 1 [file jcm-11-06189-s001.zip › jcm-1923662-supplementary-3.pdf]

SUPPLEMENTARY MATERIAL FOR:

Hospitalisation is prognostic of survival in chronic thromboembolic pulmonary hypertension

Pavel Jansa[1], David Ambrož[1], Michael Aschermann[1], Vladimír Černý[2], Vladimír Dytrych[1], Samuel Heller[1], Jan Kunstýř[3], Jaroslav Lindner[4], Aleš Linhart[1], Matúš Nižnanský[4], Michal Paďour[1], Tomáš Prskavec[4], Michal Širanec[1], Susan Edwards[5], Virginie Gressin[5], Matyáš Kuhn[6], Lilla Di Scala[5]

Table S1 Baseline characteristics of not-operated patients at landmark time-points

	Month 3 landmark		Month 6 landmark		Month 9 landmark		Month 12 landmark	
**Parameter**	**Prior hospitalisation event (n=29)**	**No prior hospitalisation event (n=178)**	**Prior hospitalisation event (n=35)**	**No prior hospitalisation event (n=164)**	**Prior hospitalisation event (n=44)**	**No prior hospitalisation event (n=151)**	**Prior hospitalisation event (n=50)**	**No prior hospitalisation event (n=141)**
**Age, mean (SD), years**	67.8 (8.66)	66.2 (12.99)	66.8 (10.78)	66.2 (13.04)	66.0 (10.66)	66.4 (13.19)	65.8 (11.24)	66.5 (13.28)
**Sex, n (%), female**	19 (65.5%)	93 (52.2%)	22 (62.9%)	86 (52.4%)	23 (52.3%)	84 (55.6%)	27 (54.0%)	79 (56.0%)
**BMI, mean (SD), kg/m^2 [n]**	29.6 (7.04) [25]	28.6 (5.57) [159]	28.8 (5.33) [30]	28.7 (5.64) [147]	29.3 (5.35) [39]	28.6 (5.65) [134]	29.5 (5.11) [45]	28.7 (5.64) [125]
**DVT history, n (%)**	7 (25.0%)	72 (41.1%)	11 (33.3%)	66 (40.7%)	13 (31.0%)	61 (40.9%)	17 (35.4%)	56 (40.3%)
**PE history, n (%)**	23 (79.3%)	131 (73.6%)	23 (65.7%)	124 (75.6%)	31 (70.5%)	112 (74.2%)	37 (74.0%)	105 (74.5%)

**Time from first PE to diagnosis, mean (SD), years**	5.8 (6.80)	5.3 (7.40)	4.9 (6.15)	5.3 (7.55)	6.2 (6.72)	5.0 (7.51)	5.8 (6.53)	5.2 (7.66)
**NYHA FC, n (%) [n]**								
FC I/II	1 (3.7%) [27]	13 (7.8%) [166]	1 (3.1%) [32]	13 (8.5%) [153]	1 (2.4%) [41]	12 (8.5%) [141]	1 (2.1%) [47]	12 (9.2%) [131]
FC III/IV	26 (96.3%) [27]	153 (92.2%) [166]	31 (96.9%) [32]	140 (91.5%) [153]	40 (97.6%) [41]	129 (91.5%) [141]	46 (97.9%) [47]	119 (90.8%) [131]
**6MWT, mean (SD), m [n]**	273.6 (114.1) [22]	338.9 (118.3) [138]	284.6 (125.6) [25]	342.4 (116.5) [128]	290.7 (127.4) [35]	344.4 (114.5) [117]	301.2 (127.9) [41]	348.0 (113.4) [107]
**RHC**								
mPAP, mean (SD), mmHg [n]	49.2 (13.89) [28]	42.4 (11.78) [173]	47.8 (13.98) [34]	42.0 (11.46) [160]	47.8 (13.02) [42]	41.4 (11.46) [148]	47.5 (13.02) [48]	40.9 (11.35) [138]
PVR, mean (SD), dyn×s/cm^5 [n]	841.5 (399.3) [27]	596.9 (301.5) [171]	749.3 (394.0) [33]	586.9 (291.2) [158]	693.8 (350.8) [41]	580.8 (292.5) [146]	665.6 (331.7) [47]	574.0 (291.8) [137]
CI, mean (SD), L/min/m^2 [n]	2.1 (0.46) [27]	2.4 (0.55) [169]	2.3 (0.56) [33]	2.4 (0.55) [156]	2.3 (0.55) [41]	2.4 (0.53) [144]	2.4 (0.61) [47]	2.4 (0.51) [134]
**BNP, mean (SD), pg/ml [n]**	463.7 (460.1) [12]	271.4 (327.2) [86]	329.5 (285.7) [17]	272.1 (336.0) [80]	286.4 (258.6) [23]	281.3 (349.6) [73]	286.5 (330.3) [26]	241.8 (271.9) [67]
**Anticoagulation, n (%) [n]**								
NOAC	1 (3.4%) [29]	7 (3.9%) [178]	1 (2.9%) [35]	7 (4.3%) [164]	1 (2.3%) [44]	7 (4.6%) [151]	1 (2.0%) [50]	7 (5.0%) [141]
Vitamin K antagonist	21 (72.4%) [29]	156 (87.6%) [178]	27 (77.1%) [35]	144 (87.8%) [164]	37 (84.1%) [44]	131 (86.8%) [151]	43 (86.0%) [50]	122 (86.5%) [141]
Other anticoagulants	28 (96.6%) [29]	171 (96.1%) [178]	34 (97.1%) [35]		43 (97.7%) [44]		49 (98.0%) [50]	

				157 (95.7%) [164]		144 (95.4%) [151]		134 (95.0%) [141]
**Time to diagnosis from study start*, mean (SD), years**	7.92 (3.390)	7.94 (3.132)	7.81 (3.218)	8.03 (3.157)	7.57 (2.920)	8.04 (3.216)	7.85 (2.949)	7.95 (3.266)

*1 January 2003

6MWT, 6-minute walk test; BMI, body mass index; BNP, brain natriuretic peptide; CI, cardiac index; DVT, deep vein thrombosis; mPAP, mean pulmonary artery pressure; NOAC, non-vitamin K antagonist oral anticoagulants; NYHA FC, New York Heart Association functional class; PE, pulmonary embolism; PVR, pulmonary vascular resistance; RHC, right heart catheterisation parameters; SD, standard deviation.

Table S2 Baseline characteristics of operated patients at landmark time-points

	Month 3 landmark		Month 6 landmark		Month 9 landmark		Month 12 landmark	
**Parameter**	**Prior hospitalisation event (n=47)**	**No prior hospitalisation event (n=182)**	**Prior hospitalisation event (n=65)**	**No prior hospitalisation event (n=153)**	**Prior hospitalisation event (n=79)**	**No prior hospitalisation event (n=139)**	**Prior hospitalisation event (n=85)**	**No prior hospitalisation event (n=130)**
**Age, mean (SD), years**	62.7 (11.04)	59.1 (12.17)	61.2 (11.93)	58.9 (12.25)	59.7 (12.47)	59.5 (12.04)	59.0 (13.42)	60.0 (11.42)
**Sex, n (%), female**	18 (38.3%)	68 (37.4%)	29 (44.6%)	54 (35.3%)	36 (45.6%)	47 (33.8%)	40 (47.1%)	42 (32.3%)
**BMI, mean (SD), kg/m^2 [n]**	27.6 (5.13) [41]	28.1 (5.19) [154]	27.9 (4.98) [53]	28.1 (5.25) [134]	28.1 (4.88) [65]	28.0 (5.32) [122]	28.2 (4.97) [72]	27.9 (5.25) [113]
**DVT history, n (%)**	23 (51.1%)	74 (40.7%)	29 (45.3%)	64 (41.8%)	34 (43.6%)	59 (42.4%)	37 (44.0%)	55 (42.3%)
**PE history, n (%)**	37 (78.7%)	147 (80.8%)	54 (83.1%)	123 (80.4%)	62 (78.5%)	115 (82.7%)	68 (80.0%)	107 (82.3%)
**Time from first PE to diagnosis, mean (SD), years**	7.5 (9.48)	4.7 (6.91)	5.8 (6.86)	4.7 (7.24)	5.9 (8.22)	4.6 (6.44)	6.0 (8.09)	4.5 (6.47)
**NYHA FC, n (%) [n]**								
FC I/II	1 (2.1%) [47]	21 (11.6%) [181]	4 (6.2%) [65]	17 (11.2%) [152]	4 (5.1%) [79]	17 (12.3%) [138]	5 (6.0%) [84]	16 (12.3%) [130]
FC III/IV	46 (97.9%) [47]	160 (88.4%) [181]	61 (93.8%) [65]	135 (88.8%) [152]	75 (94.9%) [79]	121 (87.7%) [138]	79 (94.0%) [84]	114 (87.7%) [130]

**6MWT, mean (SD), m [n]**	313.7 (94.16) [38]	358.7 (99.55) [161]	313.3 (89.67) [56]	369.7 (98.35) [133]	318.4 (90.23) [69]	372.9 (98.78) [120]	322.9 (91.99) [73]	373.8 (98.65) [113]
**RHC**								
mPAP, mean (SD), mmHg [n]	53.1 (12.09) [47]	50.3 (11.94) [175]	52.5 (11.99) [63]	50.1 (11.92) [148]	52.2 (11.80) [77]	50.0 (12.02) [134]	51.9 (12.02) [82]	49.9 (11.79) [126]
PVR, mean (SD), dyn×s/cm^5 [n]	805.9 (285.5) [47]	798.7 (353.3) [175]	841.8 (338.2) [63]	783.3 (338.1) [148]	826.1 (322.6) [77]	786.2 (347.4) [134]	825.6 (336.3) [81]	776.3 (319.3) [127]
CI, mean (SD), L/min/m^2 [n]	2.2 (0.44) [46]	2.2 (0.53) [171]	2.2 (0.46) [62]	2.2 (0.51) [144]	2.2 (0.45) [76]	2.2 (0.52) [130]	2.2 (0.47) [81]	2.2 (0.51) [122]
**BNP, mean (SD), pg/ml [n]**	379.1 (247.7) [12]	460.5 (681.1) [76]	289.8 (241.0) [16]	472.3 (685.9) [68]	356.3 (375.6) [24]	470.1 (705.4) [60]	316.9 (319.3) [25]	475.5 (716.5) [58]
**Anticoagulation, n (%) [n]**								
NOAC, n (%)	–	4 (2.2%) [179]	–	4 (2.6%) [153]	–	4 (2.9%) [139]	–	4 (3.1%) [130]
Vitamin K antagonist	37 (78.7%) [47]	151 (84.4%) [179]	56 (86.2%) [65]	126 (82.4%) [153]	67 (84.8%) [79]	115 (82.7%) [139]	73 (85.9%) [85]	106 (81.5% [130]
Other anticoagulants, n (%)	47 (100%) [47]	175 (97.8%) [179]	65 (100%) [65]	149 (97.4%) [153]	79 (100%) [79]	135 (97.1%) [139]	85 (100%) [85]	126 (96.9%) [130]

**Time to diagnosis from study start*, mean (SD), years**	6.48 (3.357)	7.27 (3.488)	5.88 (3.129)	7.65 (3.468)	6.20 (3.295)	7.64 (3.453)	6.24 (3.516)	7.70 (3.349)

*1 January 2003

6MWT, 6-minute walk test; BMI, body mass index; BNP, brain natriuretic peptide; CI, cardiac index; DVT, deep vein thrombosis; mPAP, mean pulmonary artery pressure; NOAC, non-vitamin K antagonist oral anticoagulants; NYHA FC, New York Heart Association functional class; PE, pulmonary embolism; PVR, pulmonary vascular resistance; RHC, right heart catheterisation parameters; SD, standard deviation.

Table S3. Cause of death in study cohort, by operability status

Reason for death, n (%)	Operable (n=104)	Inoperable (n=61)	Total (n=165)
Pulmonary embolism	27 (26.0%)	21 (34.4%)	48 (29.1%)
Ischaemic Heart Disease	14 (13.5%)	10 (16.4%)	24 (14.5%)
Other	11 (10.6%)	9 (14.8%)	20 (12.1%)
Malignancy	12 (11.5%)	5 (8.2%)	17 (10.3%)
Chronic obstructive pulmonary disease	8 (7.7%)	5 (8.2%)	13 (7.9%)
Infection	9 (8.7%)	3 (4.9%)	12 (7.3%)
Heart failure	8 (7.7%)	3 (4.9%)	11 (6.7%)
Renal failure	5 (4.8%)	0	5 (3.0%)
Stroke (haemorrhagic)	5 (4.8%)	0	5 (3.0%)
Arrythmia	2 (1.9%)	2 (3.3%)	4 (2.4%)
Haemorrhage	1 (1.0%)	1 (1.6%)	2 (1.2%)
Gastric ulcer	0	1 (1.6%)	1 (0.6%)
Injury	0	1 (1.6%)	1 (0.6%)
Respiratory failure	1 (1.0%)	0	1 (0.6%)
Stroke (ischaemic)	1 (1.0%)	0	1 (0.6%)

Data are based on International Classification of Disease (ICD) codes extracted from the national death registry.

Table S4. Cause of death in study cohort, by whether patients were operated on

**Reason for death, n (%)**	**Operated (n=61)**	**Not operated (n=104)**	**Total (n=165)**
Pulmonary embolism	16 (26.2%)	32 (30.8%)	48 (29.1%)
Ischaemic Heart Disease	6 (9.8%)	18 (17.3%)	24 (14.5%)
Other	8 (13.1%)	12 (11.5%)	20 (12.1%)
Malignancy	8 (13.1%)	9 (8.7%)	17 (10.3%)
Chronic obstructive pulmonary disease	3 (4.9%)	10 (9.6%)	13 (7.9%)
Infection	5 (8.2%)	7 (6.7%)	12 (7.3%)
Heart failure	5 (8.2%)	6 (5.8%)	11 (6.7%)
Renal failure	3 (4.9%)	2 (1.9%)	5 (3.0%)
Stroke (haemorrhagic)	4 (6.6%)	1 (1.0%)	5 (3.0%)
Arrythmia	1 (1.6%)	3 (2.9%)	4 (2.4%)
Haemorrhage	1 (1.6%)	1 (1.0%)	2 (1.2%)
Gastric ulcer	0	1 (1.0%)	1 (0.6%)
Injury	0	1 (1.0%)	1 (0.6%)
Respiratory failure	0	1 (1.0%)	1 (0.6%)
Stroke (ischaemic)	1 (1.6%)	0	1 (0.6%)

Data are based on International Classification of Disease (ICD) codes extracted from the national death registry.

Figure S1 Included patients (those who survived for at least 3 months post-diagnosis) by operability status and whether they underwent PEA

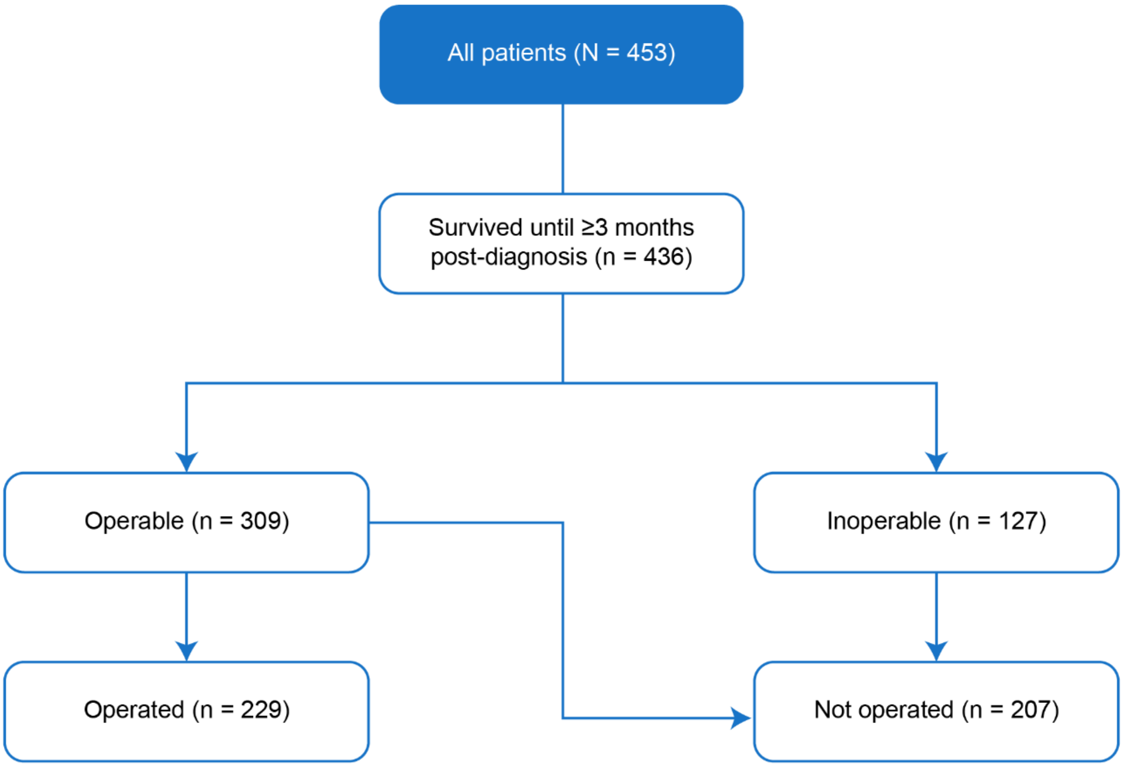

PEA, pulmonary endarterectomy

Figure S2 Landmark analysis patient disposition for (a) not-operated (b) operated patients. The number of patients listed as ‘Died’ in each group refers to the status at the data cut-off of 31 December 2018; all patients who did not die by this date were classified as ‘censored’. Percentages may not total to 100% as the denominator is number of patients, however, patients may have had more than one hospitalisation. Reasons for hospitalisation are summarised in the final row.

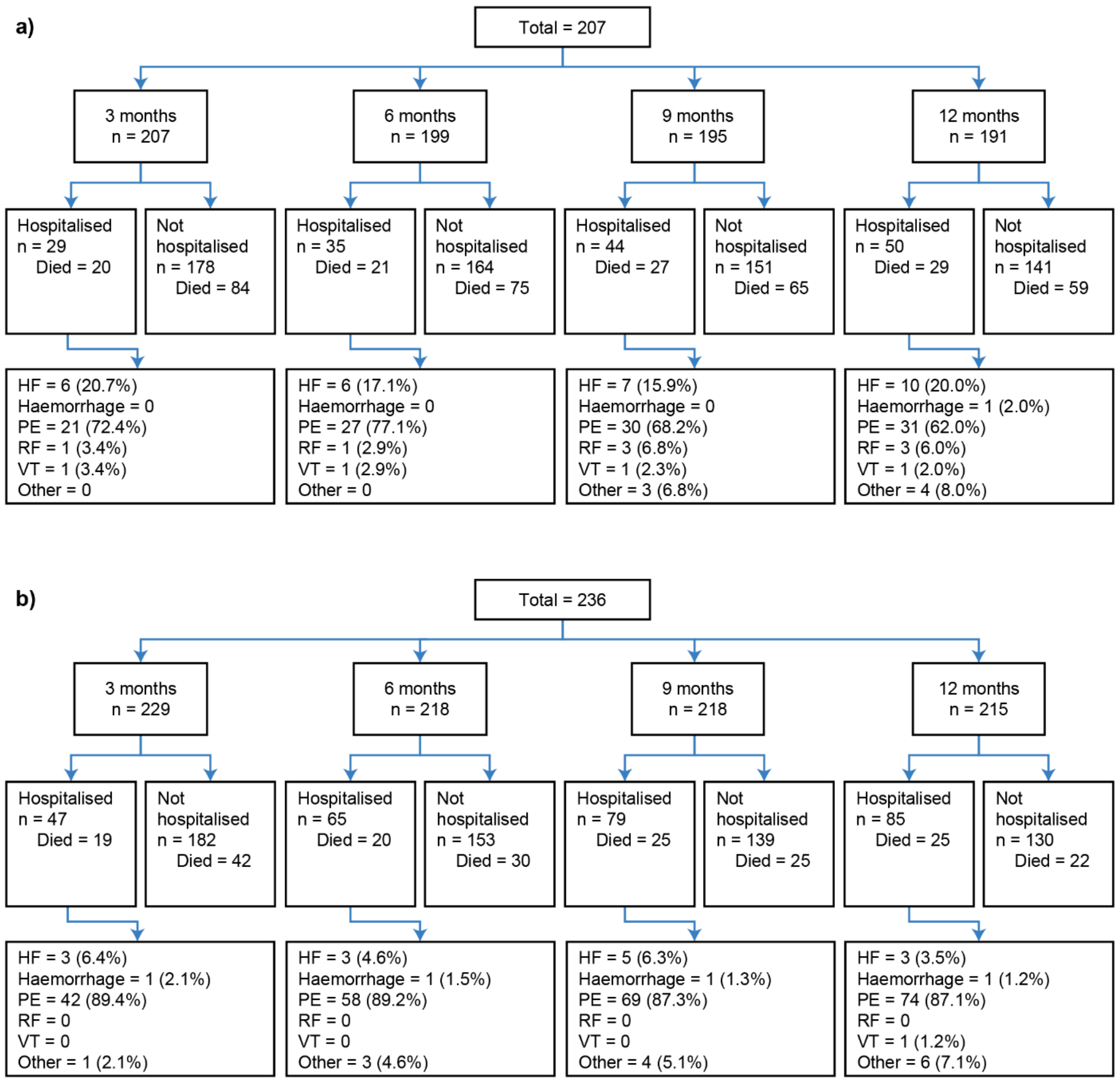

HF, heart failure; PE, pulmonary embolism; RF, respiratory failure; VT, venous thromboembolism.

Figure S3 Kaplan-Meier analysis: survival probability at each landmark time-point (model M1) in a) not-operated patients and b) operated patients.

a)

3 months

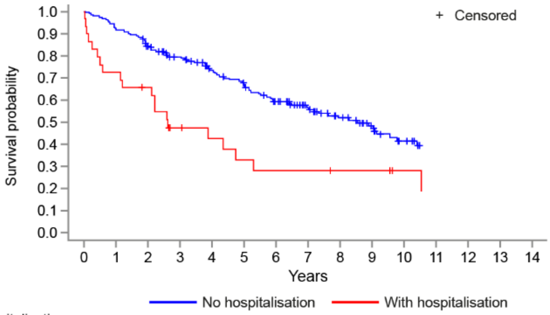

	0	1	2	3	4	5	6	7	8	9	10
**No hospitalisation**											
At risk	178	163	143	126	105	94	79	64	51	39	24
Event	0	15	28	36	45	54	65	68	73	76	81
Censored	0	0	7	16	28	30	34	46	54	63	73
**With hospitalisation**											
At risk	29	21	18	11	9	7	6	6	5	5	3
Event	0	8	10	15	16	18	19	19	19	19	19
Censored	0	0	1	3	4	4	4	4	5	5	7

6 months

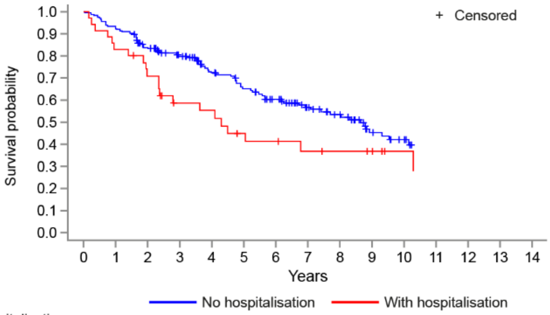

	0	1	2	3	4	5	6	7	8	9	10
**No hospitalisation**											
At risk	164	152	129	114	94	83	72	56	47	29	21
Event	0	12	27	32	42	51	57	61	64	70	72
Censored	0	0	8	18	28	30	35	47	53	65	71
**With hospitalisation**											
At risk	35	29	24	17	16	12	11	9	8	7	4
Event	0	6	10	14	15	18	19	20	20	20	20
Censored	0	0	1	4	4	5	5	6	7	8	11

9 months

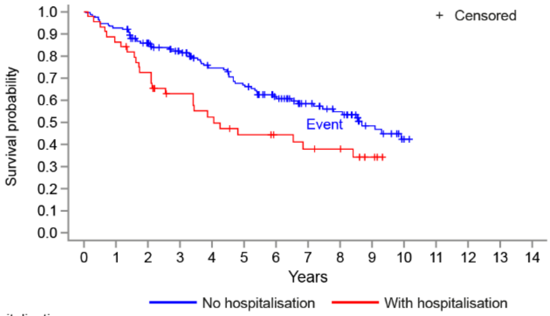

	0	1	2	3	4	5	6	7	8	9	10
**No hospitalisation**											
At risk	151	140	118	105	87	76	63	50	42	27	16
Event	0	11	21	26	35	45	51	53	56	60	63
Censored	0	0	12	20	29	30	37	48	53	64	72
**With hospitalisation**											
At risk	44	38	31	24	20	16	14	12	11	7	
Event	0	6	12	16	20	23	23	25	25	26	
Censored	0	0	1	4	4	5	7	7	8	11	

12 months

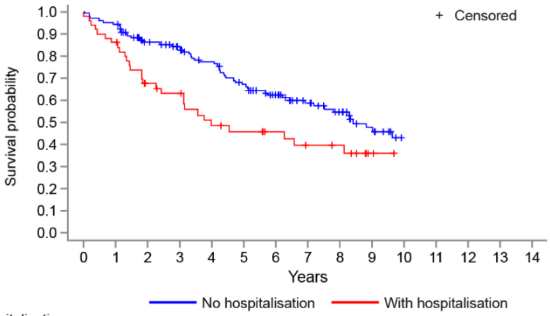

	0	1	2	3	4	5	6	7	8	9
**No hospitalisation**										
At risk	141	133	107	97	85	72	59	47	37	25
Event	0	8	19	21	29	40	45	48	51	55
Censored	0	0	15	23	27	29	37	46	53	61
**With hospitalisation**										
At risk	50	43	31	28	20	18	15	12	11	6
Event	0	7	16	18	24	25	25	27	27	28
Censored	0	0	3	4	6	7	10	11	12	16

b)

3 months

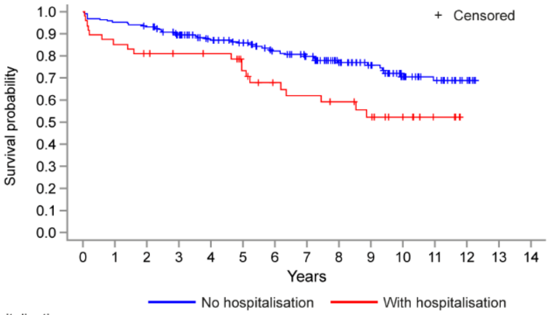

	0	1	2	3	4	5	6	7	8	9	10	11	12
**No hospitalisation**													
At risk	182	173	168	154	134	123	107	97	73	64	46	35	24
Event	0	9	13	19	23	25	30	33	36	37	41	42	42
Censored	0	0	1	9	25	34	45	52	73	81	95	105	116
**With hospitalisation**													
At risk	47	40	37	35	34	28	23	21	19	16	12	8	
Event	0	7	9	9	9	12	14	16	17	19	19	19	
Censored	0	0	1	3	4	7	10	10	11	12	16	20	

6 months

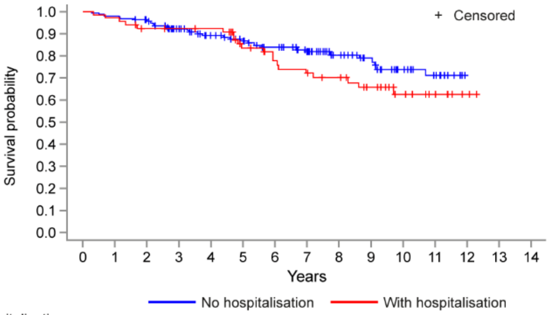

	0	1	2	3	4	5	6	7	8	9	10	11	12
**No hospitalisation**													
At risk	153	150	144	128	109	99	88	78	57	48	32	25	
Event	0	3	7	12	16	19	22	24	25	26	29	30	
Censored	0	0	2	13	28	35	43	51	71	79	92	98	
**With hospitalisation**													
At risk	65	63	58	57	56	46	40	37	34	28	20	16	8
Event	0	2	5	5	5	10	13	16	17	19	20	20	20
Censored	0	0	2	3	4	9	12	12	14	18	25	29	37

9 months

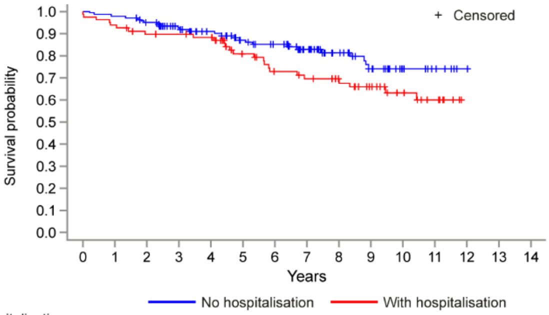

	0	1	2	3	4	5	6	7	8	9	10	11	12
**No hospitalisation**													
At risk	139	136	129	114	99	87	79	64	50	40	26	20	14
Event	0	3	7	10	12	16	18	20	21	25	25	25	25
Censored	0	0	3	15	28	36	42	55	68	74	88	94	100
**With hospitalisation**													
At risk	79	74	68	67	65	52	45	41	37	30	22	17	
Event	0	5	8	8	9	14	19	21	21	23	24	25	
Censored	0	0	3	4	5	13	15	17	21	26	33	37	

12 months

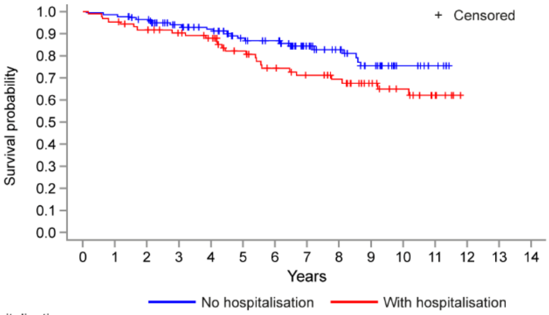

	0	1	2	3	4	5	6	7	8	9	10	11
**No hospitalisation**												
At risk	130	128	122	105	95	82	76	56	46	35	22	17
Event	0	2	5	8	10	14	15	17	18	22	22	22
Censored	0	0	3	17	25	34	39	57	66	73	86	91
**With hospitalisation**												
At risk	85	81	75	70	65	55	47	42	38	30	23	14
Event	0	4	7	8	10	14	19	21	22	23	24	25
Censored	0	0	3	7	10	16	19	22	25	32	38	46

Figure S4 Forest plot showing hazard ratios (HRs) for non-adjusted and adjusted models at landmark time-points in a) not-operated patients and b) operated patients.

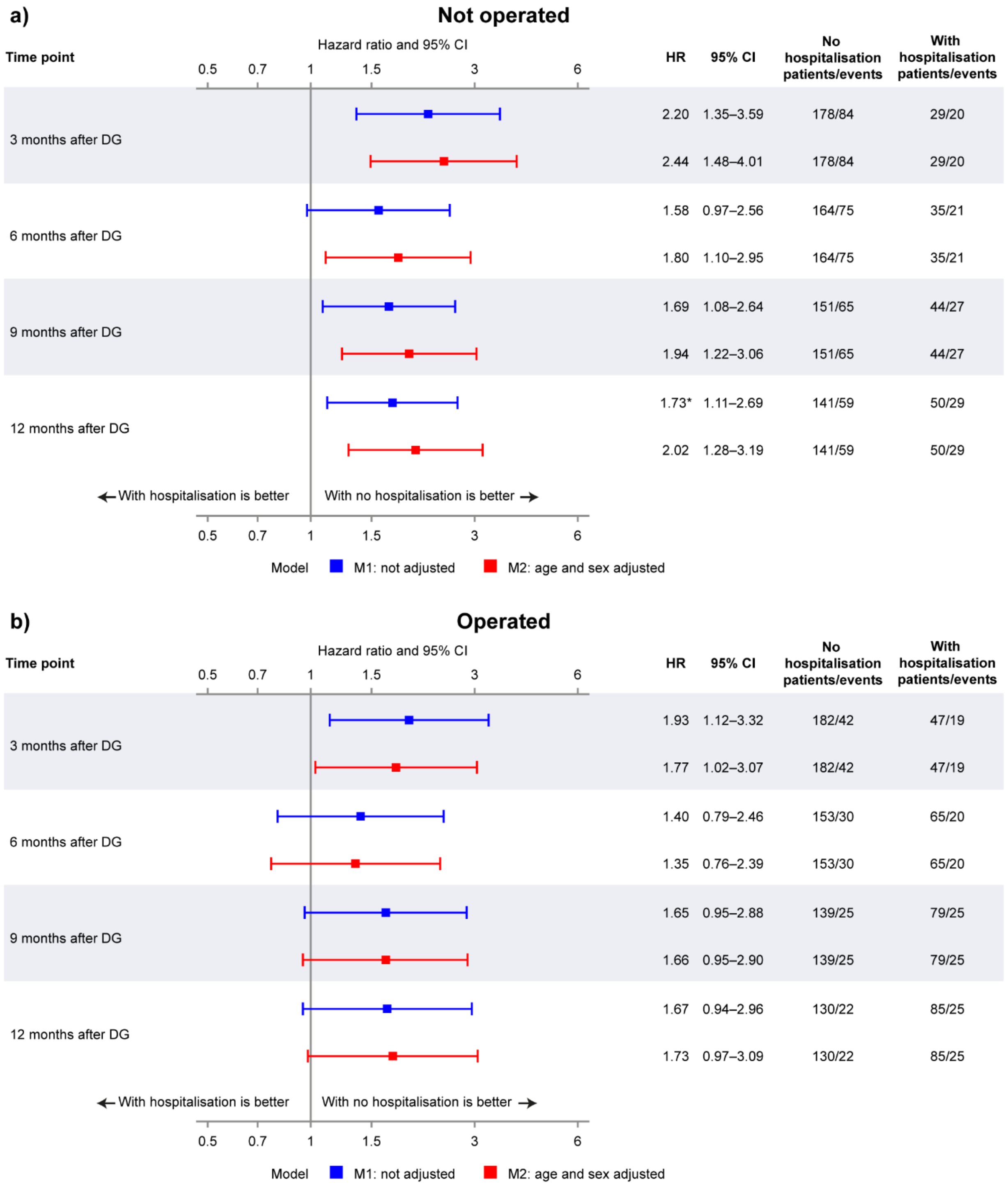

CI, confidence interval; DG, diagnosis; HR, hazard ratio.

Figure S5 Kaplan-Meier analysis in operable patients who were not-currently-operated patients: survival probability at each landmark time-point (model M1).

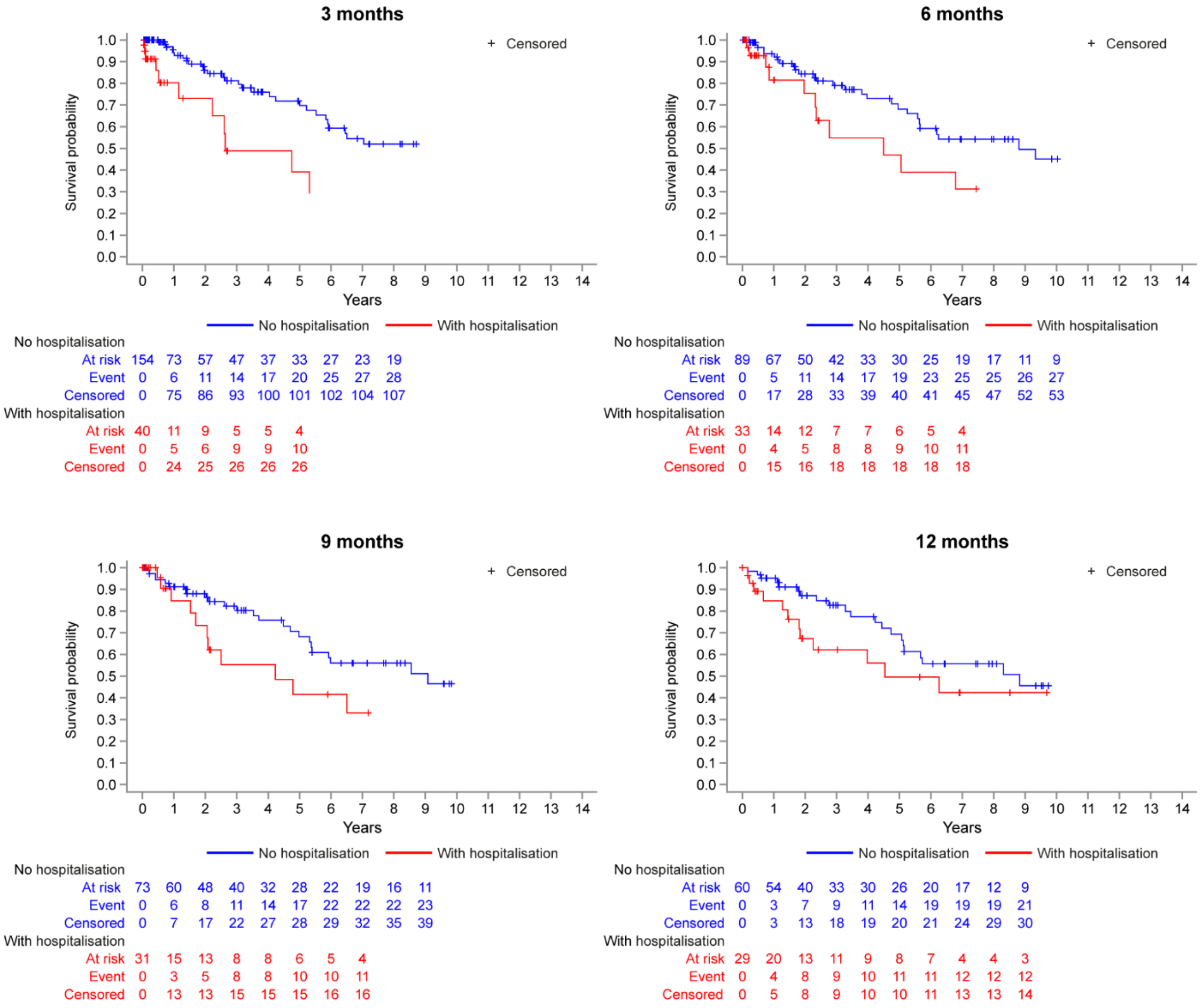

Figure S6 Forest plot showing hazard ratios (HRs) for non-adjusted and adjusted models at landmark time-points in a) operable patients who were not-currently-operated and b) operable patients who were already-operated.

a) **Operable – not-currently operated patients**

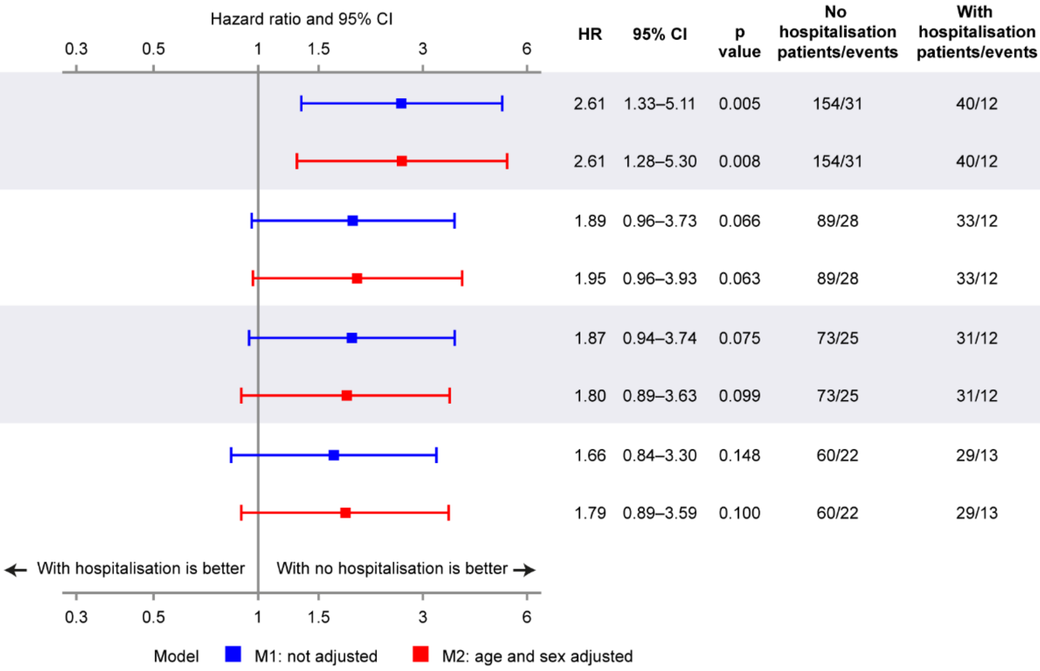

Time point	HR	95% CI	p value	No hospitalisation patients/events	With hospitalisation patients/events
3 months after DG	2.61	1.33–5.11	0.005	154/31	40/12
	2.61	1.28–5.30	0.008	154/31	40/12
6 months after DG	1.89	0.96–3.73	0.066	89/28	33/12
	1.95	0.96–3.93	0.063	89/28	33/12
9 months after DG	1.87	0.94–3.74	0.075	73/25	31/12
	1.80	0.89–3.63	0.099	73/25	31/12
12 months after DG	1.66	0.84–3.30	0.148	60/22	29/13
	1.79	0.89–3.59	0.100	60/22	29/13

b) **Operable – already operated patients**

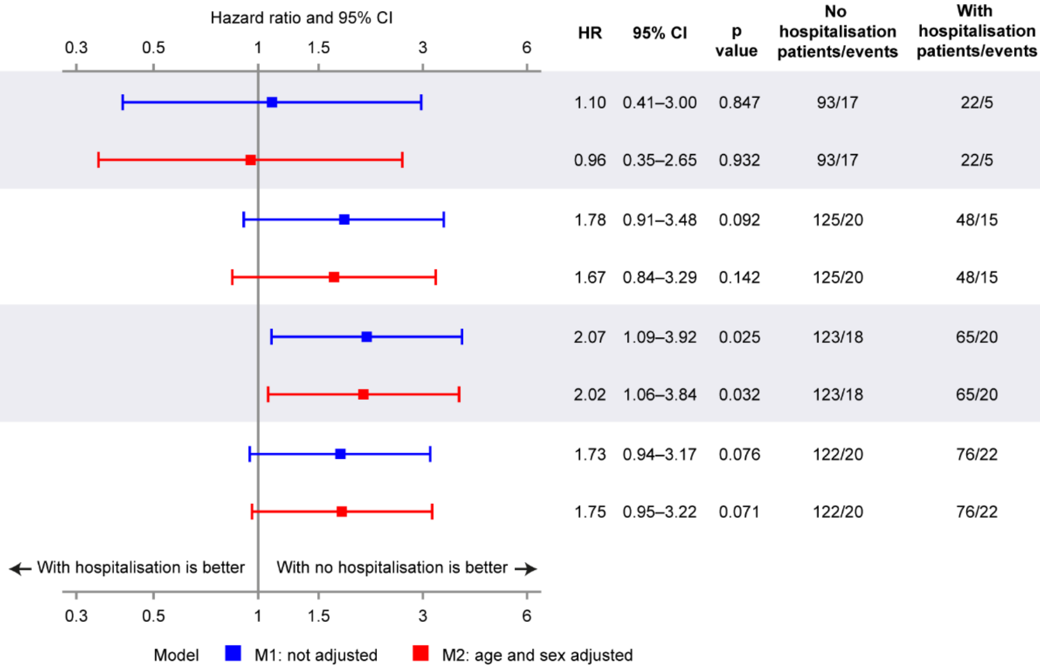

Time point	HR	95% CI	p value	No hospitalisation patients/events	With hospitalisation patients/events
3 months after DG	1.10	0.41–3.00	0.847	93/17	22/5
	0.96	0.35–2.65	0.932	93/17	22/5
6 months after DG	1.78	0.91–3.48	0.092	125/20	48/15
	1.67	0.84–3.29	0.142	125/20	48/15
9 months after DG	2.07	1.09–3.92	0.025	123/18	65/20
	2.02	1.06–3.84	0.032	123/18	65/20
12 months after DG	1.73	0.94–3.17	0.076	122/20	76/22
	1.75	0.95–3.22	0.071	122/20	76/22

CI, confidence interval; DG, diagnosis; HR, hazard ratio.

Figure S7 Kaplan-Meier analysis in operable patients who were already-operated patients: survival probability at each landmark time-point (model M1).

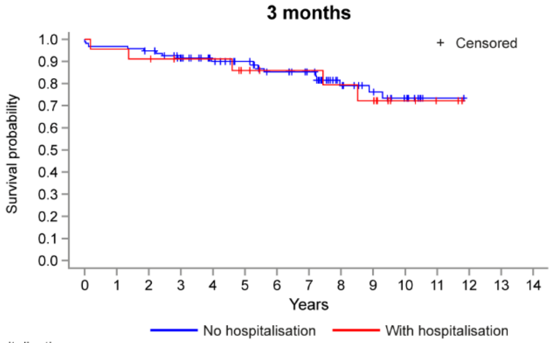

No hospitalisation												
At risk	93	90	87	78	65	60	52	48	32	27	19	10
Event	0	3	5	8	9	9	12	12	15	16	17	17
Censored	0	0	1	7	19	24	29	33	46	50	57	66
With hospitalisation												
At risk	22	21	20	18	18	15	13	13	12	10	6	4
Event	0	1	2	2	2	3	3	3	4	5	5	5
Censored	0	0	0	2	2	4	6	6	6	7	11	13

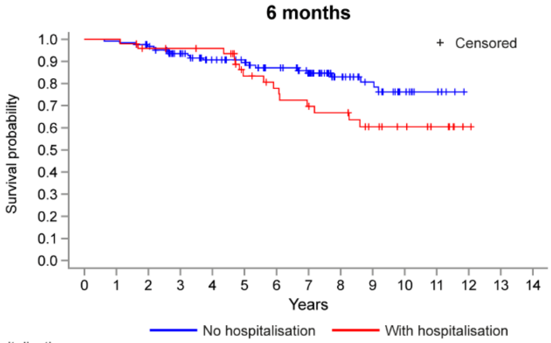

No hospitalisation													
At risk	125	124	119	105	87	81	72	64	43	36	22	17	
Event	0	1	4	8	11	12	14	16	17	18	20	20	
Censored	0	0	2	12	27	32	39	45	65	71	83	88	
With hospitalisation													
At risk	48	48	44	43	42	32	28	25	23	18	15	2	5
Event	0	0	2	2	2	7	9	12	13	15	15	15	15
Censored	0	0	2	3	4	9	11	11	12	15	18	21	28

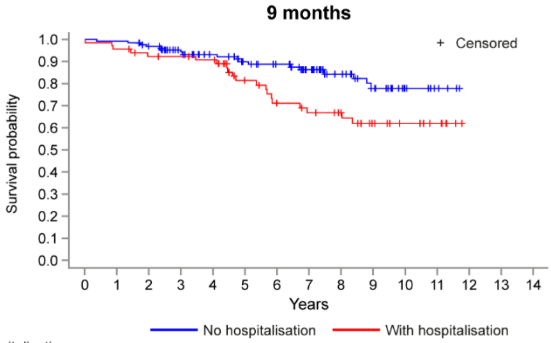

No hospitalisation												
At risk	123	122	116	101	87	78	72	58	44	35	21	16
Event	0	1	4	7	8	11	12	14	15	18	18	18
Censored	0	0	3	15	28	34	39	51	64	70	84	89
With hospitalisation												
At risk	65	62	57	56	54	41	35	31	27	21	17	14
Event	0	3	5	5	6	11	16	18	18	20	20	20
Censored	0	0	3	4	5	13	14	16	20	24	28	31

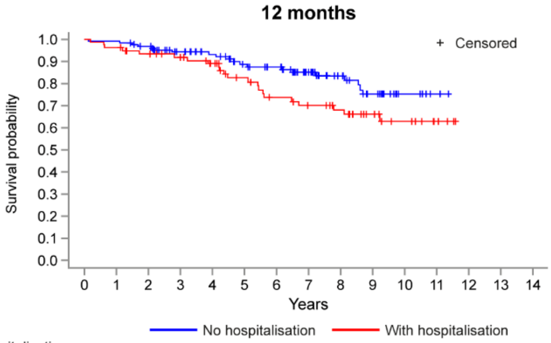

No hospitalisation												
At risk	122	121	115	98	89	78	73	53	43	32	19	14
Event	0	1	4	7	8	12	13	15	16	20	20	20
Censored	0	0	3	17	25	32	36	54	63	70	83	88
With hospitalisation												
At risk	76	73	68	63	58	48	41	37	33	26	20	12
Event	0	3	5	6	8	12	17	19	20	21	22	22
Censored	0	0	3	7	10	16	18	20	23	29	34	42
